# Cucurbitacin B Causes Increased Radiation Sensitivity of Human Breast Cancer Cells via G2/M Cell Cycle Arrest

**DOI:** 10.1155/2012/601682

**Published:** 2012-05-28

**Authors:** Suwit Duangmano, Phorntip Sae-lim, Apichart Suksamrarn, Pimpicha Patmasiriwat, Frederick E. Domann

**Affiliations:** ^1^Faculty of Medical Technology, Mahidol University, Bangkok, Thailand; ^2^Free Radical and Radiation Biology Program, Department of Radiation Oncology, University of Iowa, Iowa City, IA 52242, USA; ^3^Faculty of Science, Ramkhamhaeng University, Bangkok, Thailand

## Abstract

*Purpose*. To explore the effects of cucurbitacin B on the radiation survival of human breast cancer cells and to elucidate the cellular mechanism of radiosensitization if any. *Materials and Methods*. Human breast carcinoma cell lines were treated with cucurbitacin B before irradiation with 0–10 Gy of ^137^Cs gamma rays. The effect of cucurbitacin B on cell-survival following irradiation was evaluated by colony-forming assay. Cell cycle distributions were investigated using flow cytometry. Real-time PCR and western blots were performed to investigate the expression of cell cycle checkpoints. *Results.* Cucurbitacin B inhibited breast cancer cell proliferation in a dose-dependent manner. Only MDA-MB-231 and MCF7:5C cells but not SKBR-3 cells were radiosensitized by cucurbitacin B. Flow cytometric analysis for DNA content indicated that cucurbitacin B resulted in G2/M arrest in MDA-MB-231 and MCF7:5C but not SKBR-3 cells. Moreover, Real-time PCR and western blot analysis demonstrated upregulated p21 expression before irradiation, a likely cause of the cell cycle arrest. *Conclusion*. Taken together, these findings suggest that cucurbitacin B causes radiosensitization of some breast cancer cells, and that cucurbitacin B induced G2/M arrest is an important mechanism. Therefore, combinations of cucurbitacin B with radiotherapy may be appropriate for experimental breast cancer treatment.

## 1. Introduction

**Scheme 1:**
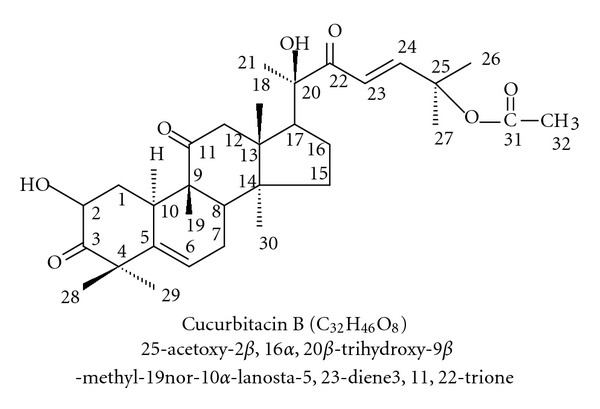
Structure of cucurbitacin B.

 Breast cancer is now the most common cause of female cancer and leading cause of cancer deaths among women in the United States and many other parts of the world [1, 2]. Over the past several decades, the incidence of breast cancer has been increasing in economically developed countries [3]. Human epidermal growth factor receptor 2 (Her2) and Estrogen receptor (ER) play critical roles in the development and progression of breast cancer. About 80% of breast cancers are hormone-receptors positive and express estrogen receptors [4]. About 20% of breast cancers do not express estrogen receptor and also Her2 [5, 6]. Therefore, breast cancer that negative for ER and Her2 does not respond to hormonal therapy. Current therapies for the treatment of breast cancer may result in drug resistance or toxicity. There is growing interest in the use of herbs to aid in the maintenance of women's health; it is interesting to use of herbs for the women's health care. Plants contain a wide variety of chemicals that have potent biological effects, including anticancer activity. Natural cucurbitacins are highly oxygenated, tetracyclic triterpenes containing the cucurbitane nucleus skeleton and are predominantly found in plants of the family cucurbitaceae, members of which have long been used in oriental medicines because of the wide range biological activity they exhibited in plants and animals. Among the various cucurbitacins, the most abundant is cucurbitacin B. Cucurbitacin B (Scheme 1) extracted from the Thai herb *Trichosanthes cucumerina *L. has been shown to have anticancer, antimicrobial, and anti-inflammatory activities [7, 8]. Several studies reported that cucurbitacin B and its relatives inhibit the growth of human malignant cells both *in vitro* and *in vivo* including breast cancer [9], head and neck squamous cell carcinoma [10], pancreatic cancer [11], hepatocellular carcinoma [12], osteosarcoma [13], and myeloid leukemia [14]. Our previous report has shown that cucurbitacin B exerts anticancer effect by inhibiting telomerase via downregulating both the* hTERT* and* c-Myc* expression and arrest of the cell cycle at G_2_/M phase in breast cancer cells [15]. Some studies have reported that cells are most sensitive to radiation in G_2_/M and most resistant in S phase [16]. For example, synchronized Chinese hamster cells were most sensitive to irradiation during mitosis and in G_2_ phase and less sensitive in G_1_ and the latter past of S phase [17, 18]. Drugs, including Docetaxel, that arrest cells cell in G_2_/M phase of cell cycle have been demonstrated as radiosensitizer agent [19]. The aims of this study were to determine the radiosensitizing potential of cucurbitacin B in human breast cancer cells and to elucidate the cellular mechanism of the radiosensitization. In the present study, we demonstrated that cucurbitacin B sensitizes human breast cancer cells to radiation by inducing them to accumulate in G_2_/M phase of the cell cycle.

## 2. Materials and Methods

### 2.1. Cell Lines and Drug Treatment

Human breast cancer cell lines (SKBR−3 (ER−/Her2+), MDA-MB-231 (ER−/Her2−), and hormone-independent MCF7:5C (ER−/Her2−)) was cultured at 37°C under a 5% CO_2_ atmosphere. SKBR-3 breast cancer cells were maintained in McCoy's 5A medium. MDA-MB-231 and MCF7:5C were maintained in DMEM/F12 medium. All medium were supplemented with 10% fetal bovine serum and 1% penicillin/streptomycin.

### 2.2. Drug and Radiation Treatment

Cucurbitacin B was authenticated by Professor Dr. Apichart suksamrarn from Faculty of Science, Ramkhamhaeng University, Bangkok, Thailand. This compound was dissolved in 10% dimethylsulfoxide (DMSO) and diluted with DMEM/F12 medium and McCoy's 5A medium to the desired concentrations prior to use. A cesium machine was used to radiate cells with a dose ranging from 0 to 10 Gy.

### 2.3. Clonogenic Survival Assay

Cells were seeded in a 100-mm culture plate and treated with the indicated concentration of cucurbitacin B for 48 hr prior to radiation exposure. After exposure, cells were then trypsinized and seed on the basis of difference density in a 60 mm culture plate with 5 mL of medium. The plates were incubated at 37°C under a 5% CO_2_ atmosphere for 14–21 day. The cells were fixed in ethanol and stained with crystal violet. Colonies containing more than 50 cells were counted as survivors. Surviving fractions were calculated by normalization to the plating efficiency of appropriate control groups.

### 2.4. Cell Cycle Analysis

For cell cycle analysis, cells were treated with cucurbitacin B at various concentrations for 48 hr and harvested. The cells were trypsinized and resuspend in 1 mL DPBS. One million cells were centrifuged and suspended in 0.5 mL of Krishan reagent (0.1% Na citrate, 0.03% NP-40, 0.05 mg/mL PI, 0.02 mg/mL RNase A) before analysis. The stained cells were subjected to DNA content/cell cycle analysis using an LSR flow cytometer.

### 2.5. Apoptosis Analysis

For apoptosis, the Annexin V-FITC Apoptosis Detection Kit (BD bioscience, Bedford, MA) was used to assess annexin V-positive cells. Briefly, fresh cell preparations were incubated with 1x annexin binding buffer and annexin V-FITC- (2.5 *μ*g/mL) conjugated primary antibody for 15 min on ice. After incubation, propidium iodide (PI; 10 *μ*g/mL) was added to the suspension and the cells were analyzed by flow cytometry using an LSR flow cytometer.

### 2.6. Quantification of *p21* mRNA

Cells (5 × 10^5^ cells/well) were seeded into 6-well plate and treated with various concentration of cucurbitacin B for 48 hr. Total RNA was isolated from each cell line using the Qiagen RNeasy Mini Kit (Qiagen, Valencia, CA). Two micrograms of total RNA were reverse-transcribed with random primer according to the manufacture's protocol using High-Capacity cDNA Reverse Transcription kit (Applied Biosystems, Foster City, CA). Real-time PCR was performed using Fast SYBR Green Master Mix (Applied Biosystems) with the Applied Biosystems 7500 Fast Real-Time PCR system (Applied Biosystems). The PCR primers set were as follows: sense5′-TGAGCCGCGACTGTGATG-3′ and anti-sense5′-GTCTCGGTGACAAAGTCGAAGTT-3′ for *p21 and* sense5′-GAAGGTGAAGGTCGGAGTC-3′ and anti-sense5′-GAAGATGGTGATGGGATTTC-3′ for *GAPDH*. The relative ratio of *p21* was then calculated using the formula: 2^−ΔΔCt^ = 2^−{ΔCt(Cucurbitacin  B-treated)−ΔCt(untreated)}^, where ΔCt = Ct(p21)−Ct(GAPDH). 

### 2.7. Western Blot Analysis

After cucurbitacin B treatment, cell pellets were collected and lysed with 100 *μ*L RIPA cell-lysis buffer (50 mM Tris pH 8.0, 150 mM NaCl, 0.1% SDS, 0.5% Na Deoxycholate, 1% TX-100) plus 1 mM NaF, 10 mM NaVO_4_, 10 mM PMSF, and 1/100 protease inhibitor cocktail (Sigma). Total protein was determined using Bio-Rad protein assay (Life science, Hercules, CA). Equal amount of proteins were separated by 12.5% SDS-Polyacrylamide gels and electrotransferred onto nitrocellulose membranes and treated with anti p21 (Santa Cruz Biotechnology, Inc., Santa Cruz, CA) overnight. Equal protein loading was confirmed on all immunoblots using beta-tubulin antibody (Developmental Studies Hybridoma Bank, University of Iowa, Iowa City, IA) at a dilution 500. Goat anti-rabbit IgG (BD Transduction Laboratories, San Diego, CA) was used as a secondary antibody against all primary antibodies. Bands were visualized by chemiluminescence with ECL plus reagent (Pierce, Rockford, IL) on a Typhoon FLA 7000.

### 2.8. Statistical Analysis

All experiments were performed at least three times. Statistical analysis was performed using one-way ANOVA to compare the effect among control (without cucurbitacin) and treated cells. *P* value < 0.05 was considered statistically significant.

## 3. Results

### 3.1. Cucurbitacin B Induced Clonogenic Inhibition of Breast Cancer Cells

**Figure 1:**
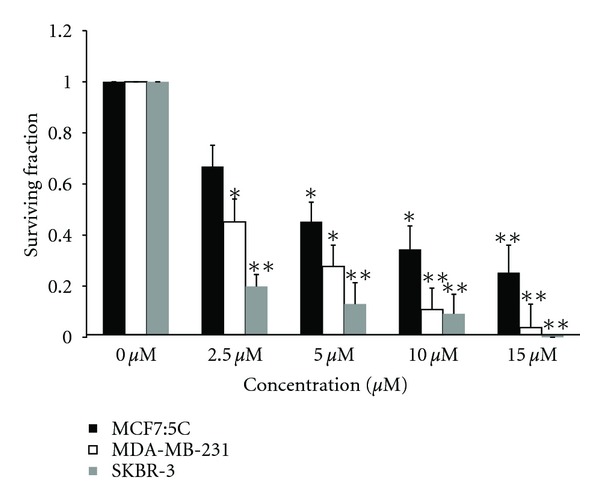
The inhibitory effects of cucurbitacin B on colony formation in breast cancer cells. MCF7:5C, MDA-MB-231, and SKBR-3 were treated with the indicated concentration of cucurbitacin B for 48 hr. After incubating, cells were seeded on the basis of difference density in a 60 mm culture plate with 5 mL of fresh medium. At 14 days after seeding, colonies were fixed and stained with 0.1% crystal violet. Results shown are the average of three independent experiments. *P < 0.05 versus nontreated control, **P < 0.01 versus nontreated control.

The inhibitory effect of cucurbitacin B on colony formation in human breast cancer cells was evaluated by clonogenic assay. Cells were incubated with cucurbitacin B alone for 48 hr and then allowed to form colonies in fresh medium. The surviving fraction as a function of drug concentration is shown in Figure 1. The average 50% (IC_50_) inhibitory concentrations for clonogenic cell death in three cells was 3.2, 2.4, and 1.9 *μ*M for MCF7:5C, and MDA-MB-231, and SKBR-3 respectively. The results are the average from three independent experiments for each cell lines. In the clonogenic assay SKBR-3, was the most sensitive cell to cucurbitacin B under the same condition for other cells.

### 3.2. Cell Cycle

**Figure 2:**
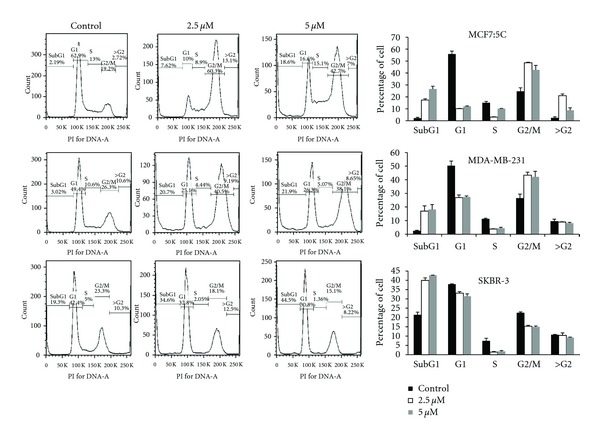
Effect of cucurbitacin B on the cell cycle progression of breast cancer cells. MCF7:5C, MDA-MB-231, and SKBR-3 were treated with cucurbitacin B for 48 hr, and then stained with propidium iodide (PI) and subjected to flow cytometric analysis. The DNA histograms shown are representative of three independent experiments. Blockage at G_2_/M and apoptotic induction was observed (SubG_1_).

Effect of cucurbitacin B on cell cycle progression in MCF7:5C, MDA-MB-231, and SKBR-3 cells were analyzed according to the principle of the DNA content in each phase of, cell cycle. Cells were treated with cucurbitacin B for 48 hr, and DNA content was analyzed via flow cytometry. MCF7:5C and MDA-MB-231 cells after treated were arrested at G_2_/M phase of cell cycle with a decrease of cells population in G1 and S phase of cell cycle, as was observed in several cancer cell lines. However, in contrast to the effect of cucurbitacin B on MCF7:5C and MDA-MB-231 cells, cucurbitacin B did not contribute to G_2_/M phase arrest in SKBR-3 cells (Figure 2).

### 3.3. Apoptosis Effect of Cucurbitacin B on Breast Cancer Cells

**Figure 3:**
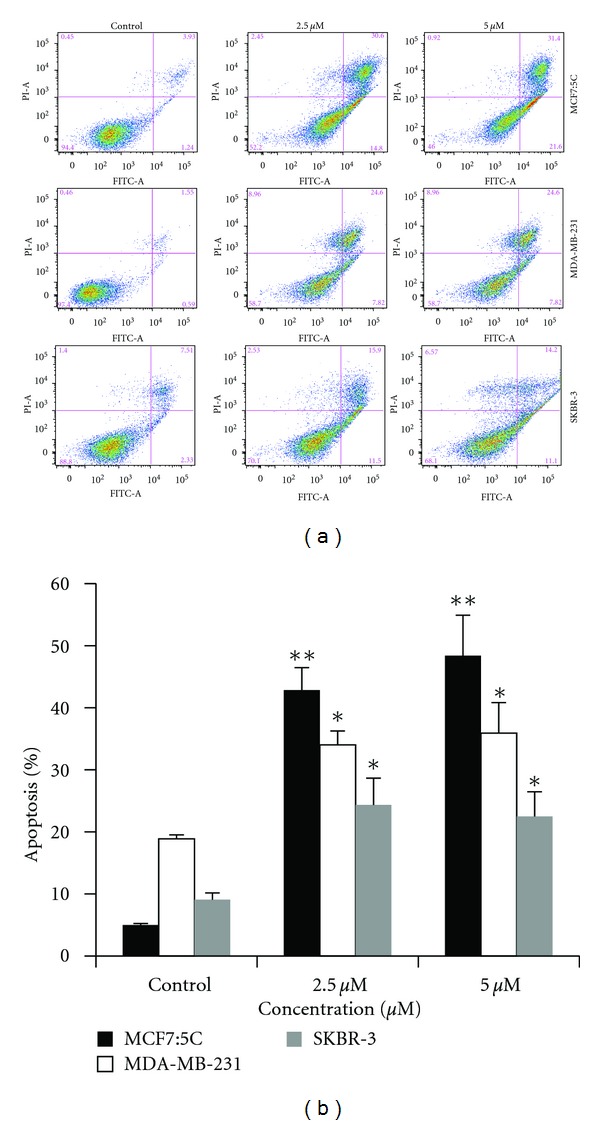
Cell death of breast cancer cells induced by cucurbitacin B. MCF7:5C, MDA-MB-231, and SKBR-3 were incubated with cucurbitacin B for 48 hr and apoptosis was analyzed by staining phosphatidylserine translocation with FITC-Annexin V. Annexin V staining is represented on the *x*-axis and PI staining is represented on the *y*-axis (a). The most representative result of three independent experiments is shown. Simple vertical bars represent the mean apoptosis rate of all of breast cancer cells (b). Results shown are the average of three independent experiments. *P < 0.05 versus nontreated control, **P < 0.01 versus nontreated control.

Apoptosis effect of cucurbitacin B was evaluated by using Annexin V-FITC and propidium iodide staining. This assay revealed that the negatively charged phospholipids phosphatidylserine found on the interior surface of the plasma membrane of the cells is trans-located to the cell surface during apoptosis. After 48 hr incubation with 0 *μ*M, 2.5 *μ*M, and 5 *μ*M of cucurbitacin B, cells were stained and subjected to bivariate flow cytometric analysis. As shown in Figure 3, untreated cells did not show any significant apoptosis, whereas cells become apoptotic with cucurbitacin B treatment at the indicated concentration in all cells.

#### 3.3.1. *p21* mRNA Expression

**Figure 4:**
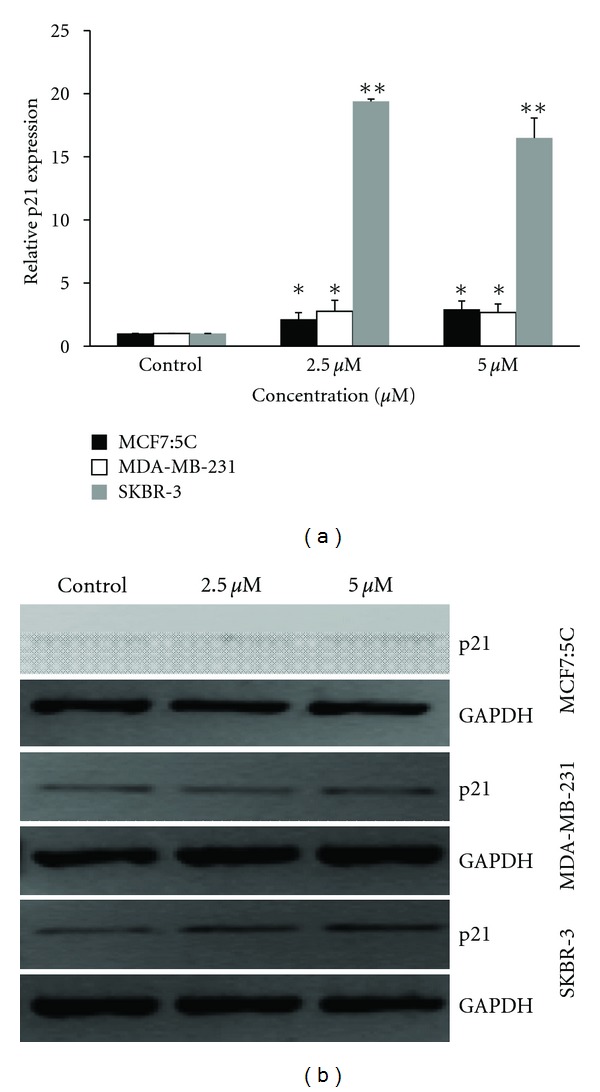
Effect of cucurbitacin B on *p21* gene expression. MCF7:5C, MDA-MB-231, and SKBR-3 were incubated for 48 hr with the specified concentrations of cucurbitacin B, and RNA was extracted for real-time PCR to quantitate the expression level of *p21*. Relative expression levels of *p21* mRNA at indicated concentration. Results shown are the average of three independent experiments. *P < 0.05 versus nontreated control, **P < 0.01 versus nontreated control.

To determine the effect of cucurbitacin B on *p21* mRNA expression, all three cell lines were incubated with cucurbitacin B for 48 hr, in parallel with untreated cell. Exposure of MCF7:5C, MDA-MB-231, and SKBR-3 cell lines to 2.5 *μ*M and 5 *μ*M of cucurbitacin B resulted in the progressive increase *p21* mRNA level. SKBR-3 shows the highest induction of p21 mRNA expression after cucurbitacin B treatment. The expression *p21* mRNA in SKBR-3 was increased up to 20 times when compared with untreated cell while MCF7:5C and MDA-MB-231 was increased 3-4 times as shown in Figure 4(a). The real-time PCR products were applied on 0.8% agarose gel containing ethidium bromide (EtBr) to scrutinize that the PCR reaction was specific and that cucurbitacin B induced gene expression of *p21*.

#### 3.3.2. Upregulation of p21 Protein by Cucurbitacin B

**Figure 5:**
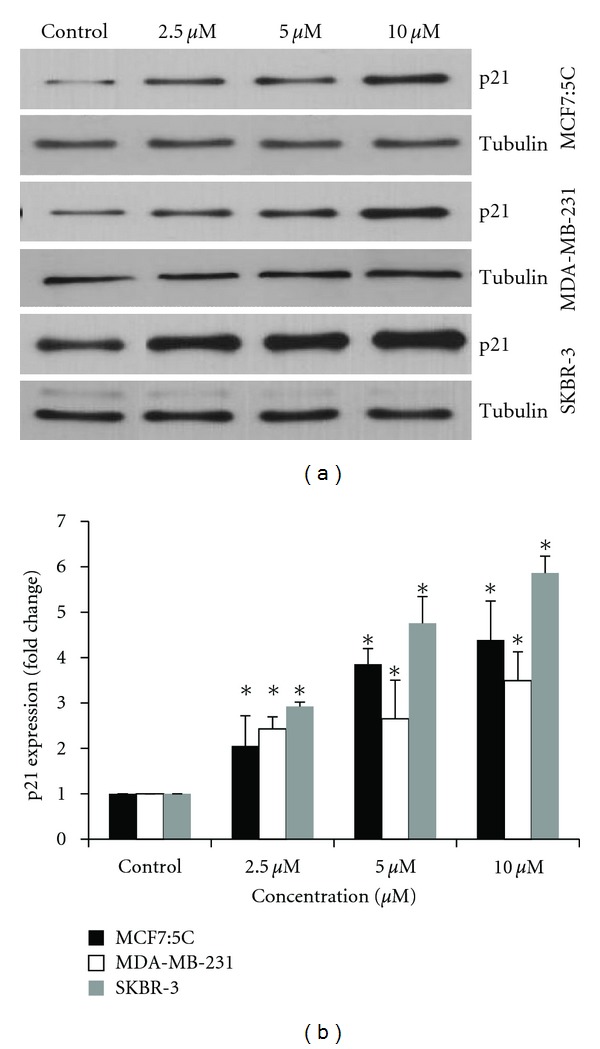
Effect of cucurbitacin B on p21 protein expression in breast cancer cells. (a) Cells were treated with cucurbitacin B for 48 hr, and then the total proteins were extracted and performed western blotting to analyze *p*21 expression. Tubulin was used as an equal loading control. (b) Densitometric analyses of expression of *p*21 relative to the untreated control. *P < 0.05 versus nontreated control, **P < 0.01 versus nontreated control.

We examined the effect of cucurbitacin B on the expression of cell cycle regulated protein by western blot analysis. Cells were incubated with the indicated concentration of cucurbitacin B for 48 hr and total protein were extracted for western blot analysis. As shown in Figure 5, protein expression of cyclin-dependent kinase inhibitor p21 was significantly increased following cucurbitacin B treatment in all study cells. SKBR-3 cells, which showed the highest mRNA accumulation in response to cucurbitacin B, also showed the greatest induction of the p21 protein; however, this was not necessarily linked to G_2_/M arrest or radiation sensitization by cucurbitacin B.

### 3.4. The Radiopotentiating Effect of CuB on Breast Cancer Cells

**Figure 6:**
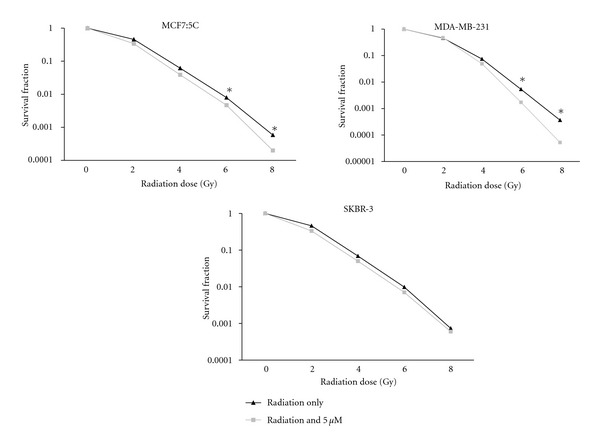
Clonogenic survival of breast cancer cells after treatment including irradiation with or without cucurbitacin B. MCF7:5C, MDA-MB-231, and SKBR-3 cells were treated with 5 *μ*M of cucurbitacin B for 48 hr before radiation. Following the incubation period with specific drug concentration, cells were harvested, resuspended in fresh medium, and then irradiated at 0–8 Gy. Colony formation was detected by 21 days later and survival curve was constructed. Data was a summary of three experiments.

To determine whether cucurbitacin B sensitized human breast cancer cells to ionizing radiation, all three cell lines were treated with 5 *μ*M cucurbitacin B for 48 hr following irradiation with a ^137^Cs gamma-irradiator at doses ranging 0–8 Gy. The cells were then allowed to form colonies in fresh medium. The plating efficiency of all cells was between 60 and 80%.  Figure 6 shows the radiation survival curves derived from clonogenic assays of the three cell lines irradiated after 48 hr incubation with cucurbitacin B. The latter slope of survival curve of MCF7:C and MDA-MB-231 cells for radiation and cucurbitacin B treated were greater than radiation only, especially at the 6 and 8 Gy radiation doses, indicating that cucurbitacin B treatment augmented the effects of radiation in both cell lines where G_2_/M arrest was observed to occur. However, SKBR-3 did not show to augment the effects of radiation, consistent with the cell cycle distribution shown in Figure 2 and the absence of a G_2_/M arrest in SKBR-3 cells.

## 4. Discussion

The Cucurbitacins are highly oxygenated, tetracyclic triterpenes containing the cucurbitane nucleus skeleton and are of great interest because of the wide range biological activity they exhibited in plants and animals. Cucurbitacins have been reported to inhibit several types of cancers. Cucurbitacins are divided into twelve categories [20]. Among the various cucurbitacins, the most abundant is cucurbitacin B [21]. Many reports have shown that cucurbitacin B has potent antiproliferative effect on breast cancer cells. For instance, cucurbitacin B, extracted from root and fruit juice of *T. cucumerina* has been reported to exert the cytotoxicity on human breast cancer cell lines [22]. Wakimoto et al. [9] reported that cucurbitacin B exerts the anticancer activity against ER−, Her2/neu amplified, and p53 mutant breast cancers both *in vitro* and *in vivo* [9]. Similarly, cucurbitacin B has been reported to inhibit Wnt signaling pathway through reduction of Wnt-associated protein and reduced translocation of galectin-3-mediated *β*-catenin to the nucleus [23].

In this study, we analyzed the anticancer activity of cucurbitacin B in human breast cancer cells lines: MCF7:5C, MDA-MB-231, and SKBR-3 using clonogenic survival assay. Among these three cells lines, SKBR-3 express high levels of Her2/neu receptor, whereas MCF7:5C and MDA-MB-231 do not express these receptor. We showed that cucurbitacin B has potent antiproliferation activity in all cell types. SKBR-3 is the most sensitive to cucurbitacin B when compared with other two cell lines. We further determined the effect of cucurbitacin B on cell cycle progression and apoptosis induction in breast cancer cell lines. The results showed that apoptotic cells were induced by cucurbitacin B treatment in all cell lines, and cell cycle is arrested at the G_2_/M phase in MCF7:5C and MDA-MB-231 cells but not in SKBR-3 cells (Figure 2). Several authors have shown that the radiosensitizing effect of anticancer drug is due to cell cycle alteration. Paclitaxel, a microtubule-stabilizing drug, has been shown to enhance radiosensitivity by blocking cells in G_2_/M phase of the cell cycle [24, 25]. Since cells in the G_2_/M phase have been reported to be more radiosensitive than in other phases of cell cycle, and cucurbitacin B treatment increases the number of cells in G_2_/M phase of the cell cycle and thus enhanced the effects of radiation on breast cancer cell line. In our study, cucurbitacin B exerted the radiosensitivity when administered 5 *μ*M of cucurbitacin B before radiation on MCF7:5C and MDA-MB-231 cells. However, no radiosensitization occurred when SKBR-3 cells were exposed to cucurbitacin B at 5 *μ*M and radiation, and no accumulation of cells in the G_2_/M phase was observed prior to the start of irradiation under these conditions. p21 and cell cycle checkpoints were shown to modulate the nucleotide excision repair process to facilitate the repair of DNA damage even in the absence of wild-type p53 [26]. Our results indicate that p21 expression level by cucurbitacin B treatment mostly showed upregulation in all cell lines especially in SKBR-3 which showed the highest induction when compared with other cell types (Figures 4 and 5). Therefore, no radiosensitization was manifest using 5 *μ*M cucurbitacin B in SKBR-3 likely because cucurbitacin B did not induce a G_2_/M cell cycle arrest. Taken together, the radiosensitizing effect by cucurbitacin B was dependent on the induction of G_2_/M arrest in breast cancer cells but not necessarily on the induction of p21. In summary, cucurbitacin B can enhance the effect of radiosensitization on breast cancer cells and studies *in vivo *are required to evaluate the biological efficacy of cucurbitacin B treatment.
