# Corrigendum to “Cucurbitacin B Causes Increased Radiation Sensitivity of Human Breast Cancer Cells via G2/M Cell Cycle Arrest”

**DOI:** 10.1155/2015/486850

**Published:** 2015-02-02

**Authors:** Suwit Duangmano, Phorntip Sae-lim, Apichart Suksamrarn, Pimpicha Patmasiriwat, Frederick E. Domann

**Affiliations:** ^1^Faculty of Medical Technology, Mahidol University, Bangkok, Thailand; ^2^Free Radical and Radiation Biology Program, Department of Radiation Oncology, University of Iowa, Iowa City, IA 52242, USA; ^3^Faculty of Science, Ramkhamhaeng University, Bangkok, Thailand

In Figure 3(a) of the published paper entitled “Cucurbitacin B Causes Increased Radiation Sensitivity of Human Breast Cancer Cells via G2/M Cell Cycle Arrest,” we mistakenly used the same flow cytometry data panel for the MDA-MB-231 cells treated with 2.5 *μ*M and 5 *μ*M cucurbitacin B. The corrected figure and legend are presented here.

## Figures and Tables

**Figure 3:**
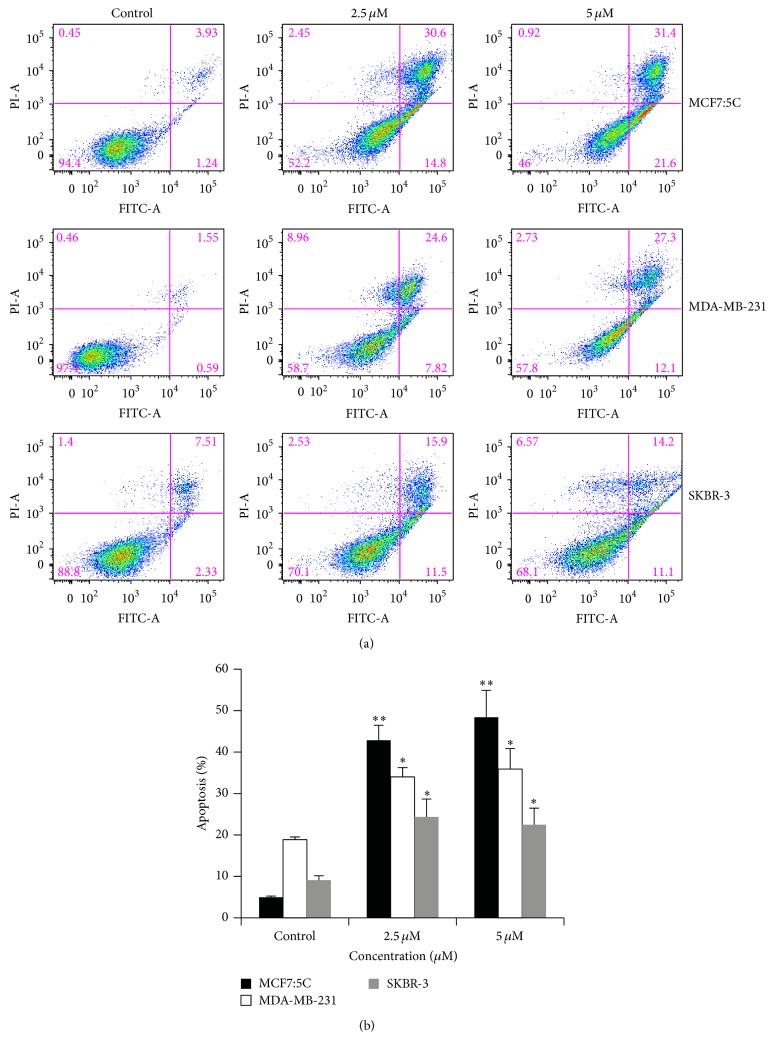
Cell death of breast cancer cells induced by cucurbitacin B. (a) MCF7:5C, MDA-MB-231, and SKBR-3 were incubated with the indicated doses of cucurbitacin B for 48 hr and apoptosis was analyzed by staining phosphatidylserine translocation with FITC-Annexin V. Annexin V staining is represented on the *x*-axis and PI staining is represented on the *y*-axis. The most representative result of three independent experiments is shown. 5 *μ*M data for MDA-MB-231 are not shown. Simple vertical bars represent the mean apoptosis rate of all of breast cancer cells (b). Results shown are the average of three independent experiments. ^*^ *P* < 0.05 versus nontreated control; ^**^ *P* < 0.01 versus nontreated control.

